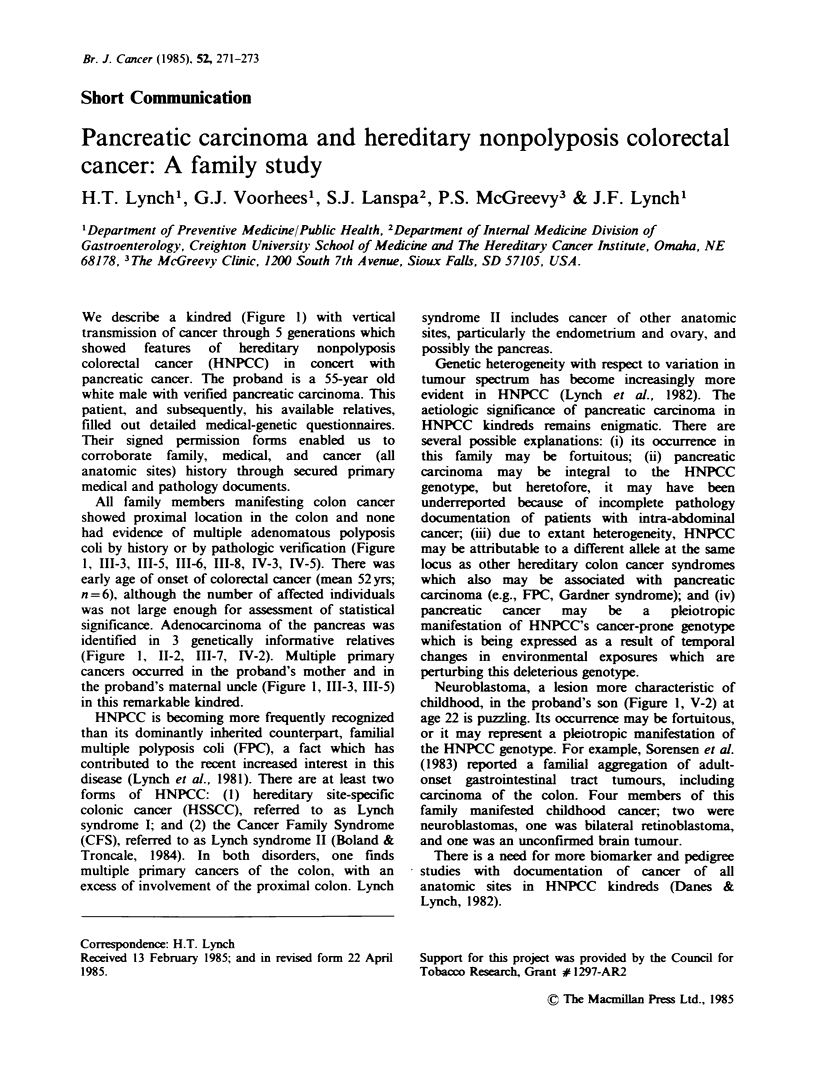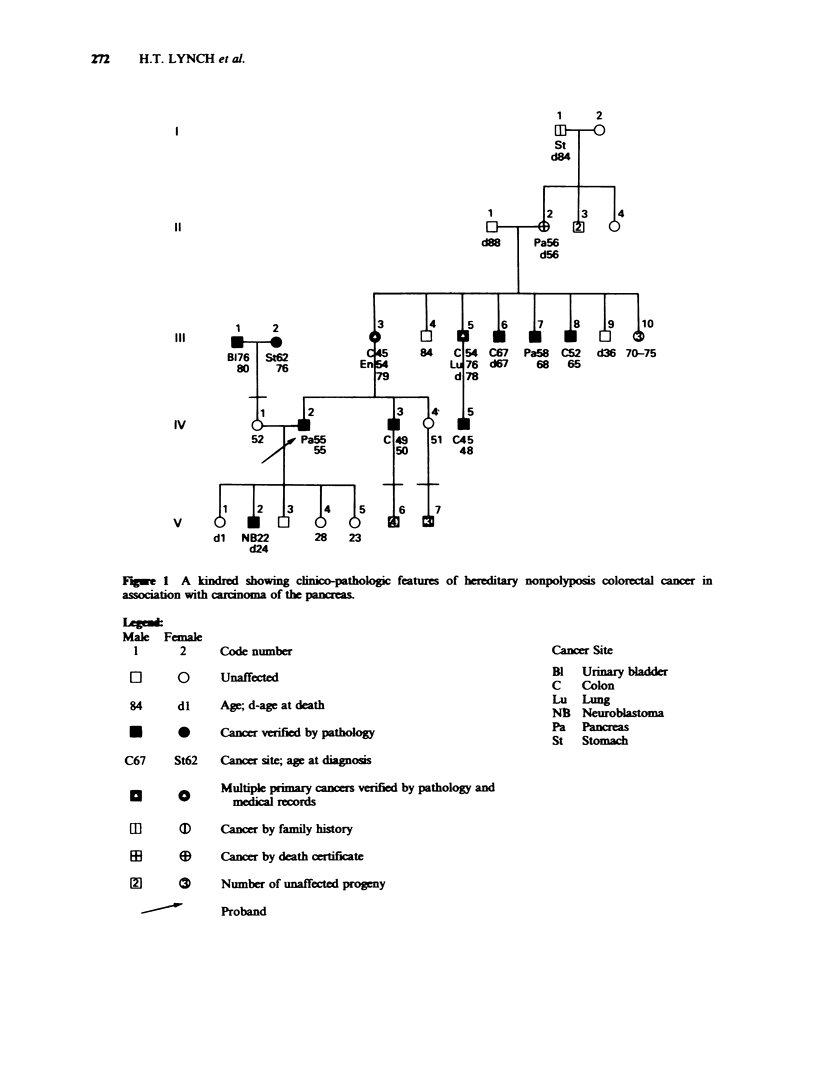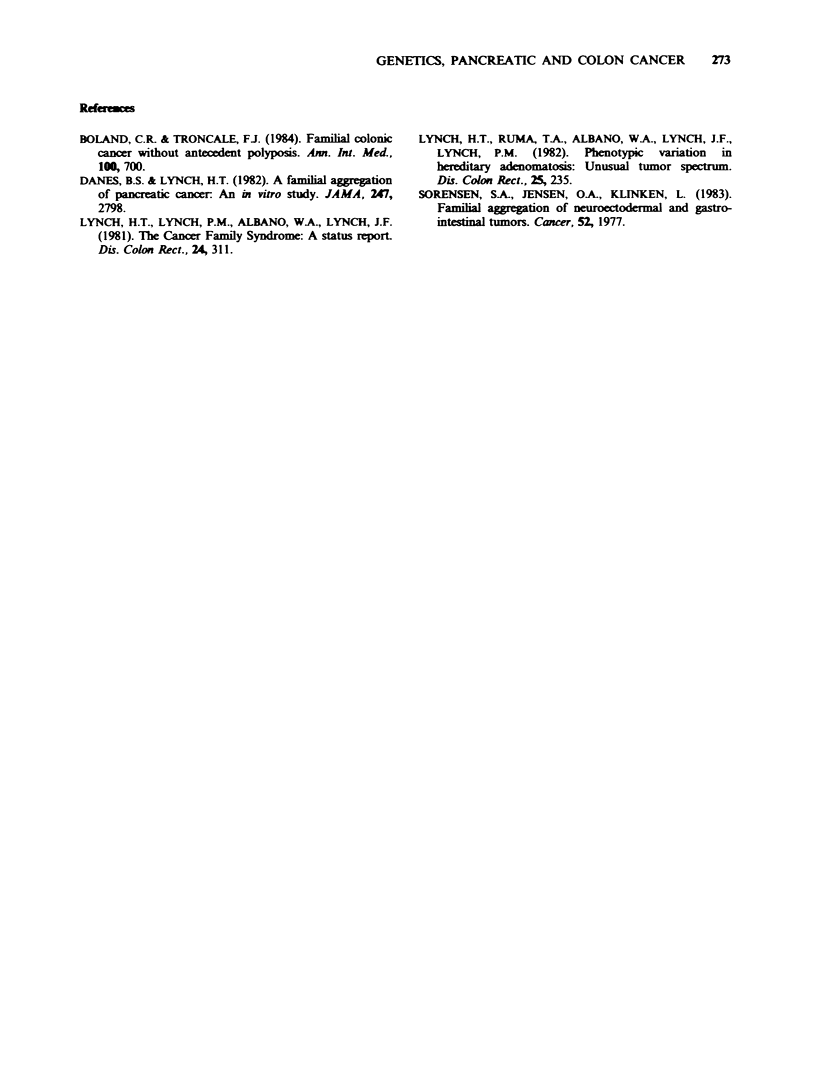# Pancreatic carcinoma and hereditary nonpolyposis colorectal cancer: a family study.

**DOI:** 10.1038/bjc.1985.187

**Published:** 1985-08

**Authors:** H. T. Lynch, G. J. Voorhees, S. J. Lanspa, P. S. McGreevy, J. F. Lynch


*Br. J. Cancer* (1985), **52**, 271–273

### Short Communication

### Pancreatic carcinoma and hereditary nonpolyposis colorectal cancer: A family study

H.T. Lynch[1], G.J. Voorhees[1], S.J. Lanspa[2], P.S. McGreevy[3] & J.F. Lynch[1]

[1]*Department of Preventive Medicine/Public Health,* [2]*Department of Internal Medicine Division of Gastroenterology, Creighton University School of Medicine and The Hereditary Cancer Institute, Omaha, NE 68178,* [3]*The McGreevy Clinic, 1200 South 7th Avenue, Sioux Falls, SD 57105, USA.*

We describe a kindred (Figure 1) with vertical transmission of cancer through 5 generations which showed features of hereditary nonpolyposis colorectal cancer (HNPCC) in concert with pancreatic cancer. The proband is a 55-year old white male with verified pancreatic carcinoma. This patient, and subsequently, his available relatives, filled out detailed medical-genetic questionnaires. Their signed permission forms enabled us to corroborate family, medical, and cancer (all anatomic sites) history through secured primary medical and pathology documents.

All family members manifesting colon cancer showed proximal location in the colon and none had evidence of multiple adenomatous polyposis coli by history or by pathologic verification (Figure 1, III-3, III-5, III-6, III-8, IV-3, IV-5). There was early age of onset of colorectal cancer (mean 52 yrs; $n=6$), although the number of affected individuals was not large enough for assessment of statistical significance. Adenocarcinoma of the pancreas was identified in 3 genetically informative relatives (Figure 1, II-2, III-7, IV-2). Multiple primary cancers occurred in the proband's mother and in the proband's maternal uncle (Figure 1, III-3, III-5) in this remarkable kindred.

HNPCC is becoming more frequently recognized than its dominantly inherited counterpart, familial multiple polyposis coli (FPC), a fact which has contributed to the recent increased interest in this disease (Lynch *et al.*, 1981). There are at least two forms of HNPCC: (1) hereditary site-specific colonic cancer (HSSCC), referred to as Lynch syndrome I; and (2) the Cancer Family Syndrome (CFS), referred to as Lynch syndrome II (Boland & Troncale, 1984). In both disorders, one finds multiple primary cancers of the colon, with an excess of involvement of the proximal colon. Lynch syndrome II includes cancer of other anatomic sites, particularly the endometrium and ovary, and possibly the pancreas.

Genetic heterogeneity with respect to variation in tumour spectrum has become increasingly more evident in HNPCC (Lynch *et al.*, 1982). The aetiologic significance of pancreatic carcinoma in HNPCC kindreds remains enigmatic. There are several possible explanations: (i) its occurrence in this family may be fortuitous; (ii) pancreatic carcinoma may be integral to the HNPCC genotype, but heretofore, it may have been underreported because of incomplete pathology documentation of patients with intra-abdominal cancer; (iii) due to extant heterogeneity, HNPCC may be attributable to a different allele at the same locus as other hereditary colon cancer syndromes which also may be associated with pancreatic carcinoma (e.g., FPC, Gardner syndrome); and (iv) pancreatic cancer may be a pleiotropic manifestation of HNPCC's cancer-prone genotype which is being expressed as a result of temporal changes in environmental exposures which are perturbing this deleterious genotype.

Neuroblastoma, a lesion more characteristic of childhood, in the proband's son (Figure 1, V-2) at age 22 is puzzling. Its occurrence may be fortuitous, or it may represent a pleiotropic manifestation of the HNPCC genotype. For example, Sorensen *et al.* (1983) reported a familial aggregation of adult-onset gastrointestinal tract tumours, including carcinoma of the colon. Four members of this family manifested childhood cancer; two were neuroblastomas, one was bilateral retinoblastoma, and one was an unconfirmed brain tumour.

There is a need for more biomarker and pedigree studies with documentation of cancer of all anatomic sites in HNPCC kindreds (Danes & Lynch, 1982).

Correspondence: H.T. Lynch
Received 13 February 1985; and in revised form 22 April 1985.

Support for this project was provided by the Council for Tobacco Research, Grant #1297-AR2

© The Macmillan Press Ltd., 1985

**Figure 1** A kindred showing clinico-pathologic features of hereditary nonpolyposis colorectal cancer in association with carcinoma of the pancreas.

**Legend:**

| Male | Female | |
|---|---|---|
| 1 | 2 | Code number |
| □ | ○ | Unaffected |
| 84 | d1 | Age; d-age at death |
| ■ | ● | Cancer verified by pathology |
| C67 | St62 | Cancer site; age at diagnosis |
| ▣ | ⊙ | Multiple primary cancers verified by pathology and medical records |
| ◫ | ⦶ | Cancer by family history |
| ⊞ | ⊕ | Cancer by death certificate |
| [2] | (3) | Number of unaffected progeny |
| ——➤ | | Proband |

**Cancer Site**

| | |
|---|---|
| Bl | Urinary bladder |
| C | Colon |
| Lu | Lung |
| NB | Neuroblastoma |
| Pa | Pancreas |
| St | Stomach |

### References

BOLAND, C.R. & TRONCALE, F.J. (1984). Familial colonic cancer without antecedent polyposis. *Ann. Int. Med.*, **100**, 700.

DANES, B.S. & LYNCH, H.T. (1982). A familial aggregation of pancreatic cancer: An *in vitro* study. *JAMA*, **247**, 2798.

LYNCH, H.T., LYNCH, P.M., ALBANO, W.A., LYNCH, J.F. (1981). The Cancer Family Syndrome: A status report. *Dis. Colon Rect.*, **24**, 311.

LYNCH, H.T., RUMA, T.A., ALBANO, W.A., LYNCH, J.F., LYNCH, P.M. (1982). Phenotypic variation in hereditary adenomatosis: Unusual tumor spectrum. *Dis. Colon Rect.*, **25**, 235.

SORENSEN, S.A., JENSEN, O.A., KLINKEN, L. (1983). Familial aggregation of neuroectodermal and gastrointestinal tumors. *Cancer*, **52**, 1977.